# Soilborne wheat mosaic virus (SBWMV) 19K protein belongs to a class of cysteine rich proteins that suppress RNA silencing

**DOI:** 10.1186/1743-422X-2-18

**Published:** 2005-03-01

**Authors:** Jeannie Te, Ulrich Melcher, Amanda Howard, Jeanmarie Verchot-Lubicz

**Affiliations:** 1 Department of Entomology and Plant Pathology, Oklahoma State University, Stillwater, OK 74078, USA; 2 Department of Biochemistry and Molecular Biology, Oklahoma State University, Stillwater, OK 74078, USA

## Abstract

Amino acid sequence analyses indicate that the *Soilborne wheat mosaic virus *(SBWMV) 19K protein is a cysteine-rich protein (CRP) and shares sequence homology with CRPs derived from furo-, hordei-, peclu- and tobraviruses. Since the hordei- and pecluvirus CRPs were shown to be pathogenesis factors and/or suppressors of RNA silencing, experiments were conducted to determine if the SBWMV 19K CRP has similar activities. The SBWMV 19K CRP was introduced into the *Potato virus X *(PVX) viral vector and inoculated to tobacco plants. The SBWMV 19K CRP aggravated PVX-induced symptoms and restored green fluorescent protein (GFP) expression to GFP silenced tissues. These observations indicate that the SBWMV 19K CRP is a pathogenicity determinant and a suppressor of RNA silencing.

## Background

Viruses survive in their hosts either by evading or countering host defenses. Viral evasion is a passive mechanism by which viruses overwhelm host defenses, or invade organs or cells where the host defenses cannot reach them. The ability of a virus to counter host defenses requires an active mechanism to either bypass or disarm the host machinery. Viruses invading vertebrate hosts produce virokines and viroceptors which interact with immune response molecules to inhibit or modulate their anti-viral activities [1,2]. Recent studies have shown many viruses infecting a wide range of eukaryotic hosts encode proteins that suppress the RNA silencing, anti-viral defense response [3-6]. Silencing suppressors encoded by viruses limit degradation of viral RNAs by the RNA silencing machinery. Among plant viruses, some silencing suppressor proteins also affect symptom development and increase virus titer. The *Cucumber mosaic virus *(CMV) 2b, the *Tobacco etch virus *(TEV) HC-Pro, and the *Tomato bushy stunt virus *(TBSV) P19 [7-10] proteins are among the best studied silencing suppressors that are also pathogenicity determinants. The TBSV P19 protein was unique because it affects disease severity in a host specific manner [11,12].

Little is known about the evolution and phylogenetic relationships of silencing suppressor proteins. In particular, viruses belonging to the genera *Furo-, Hordei-, Tobra-, Peclu-, Beny-, Carla*-, and *Pomovirus *encode small cysteine-rich proteins (CRPs) near the 3' ends of their genomes, and some have been identified as both silencing suppressor proteins and pathogenicity factors. For example, the *Barley stripe mosaic virus *(BSMV; a hordeivirus) gamma b protein and the *Peanut clump virus *(PCV; a pecluvirus) 15K protein suppress RNA silencing, modulate symptom severity, and systemic virus accumulation [13-16]. The *Tobacco rattle virus *(TRV; a tobravirus) 16K CRP has been described as a pathogenicity factor and suppresses RNA silencing [17]. In complementation studies, the *Soilborne wheat mosaic virus *(SBWMV; a furovirus) 19K CRP, the BSMV gamma b protein, and the CMV 2b (which is not a CRP) protein functionally replaced the 16K CRP of TRV [15]. Since deletion of the TRV 16K CRP ORF reduced virus accumulation in plants, functional replacement by these heterologous viral ORFs indicates that these CRPs share some common function. Characterizing the functional similarities among these CRPs is crucial to understanding their evolutionary relationship. Until now the phylogenetic relationships among these CRPs are unclear [18].

This study was undertaken to characterize the SBWMV 19K CRP. SBWMV is a bipartite RNA virus and is the type member for the genus *Furovirus *[19]. RNA1 encodes the viral replicase and putative viral movement protein (MP). The viral replicase is encoded by a single large open reading frame (ORF) and is phylogenetically related to the *Tobacco mosaic virus *(TMV) replicase [20]. The 3' proximal ORF of RNA1 encodes a 37K MP that shares sequence similarity with the dianthovirus MP [21,22]. SBWMV RNA2 encodes four proteins. The 5' proximal ORF of RNA2 encodes a 25K protein from a nonAUG start codon [23] and its role in virus infection is unknown. The coat protein (CP) ORF has an opal translational termination codon and readthrough of this codon produces a large 84K protein [23]. The CP readthrough domain (RT) is required for plasmodiophorid transmission of the virus [24]. The 3' proximal ORF of RNA2 encodes a 19K CRP.

To gain insight into the role of the SBWMV 19K CRP in virus infection, amino acid sequence comparisons were conducted to determine the relatedness of the SBWMV 19K CRP to other viral CRPs. The *Potato virus X *(PVX) infectious clone was used to express the SBWMV CRP and to study its role in virus pathogenicity and suppressing RNA silencing.

## Results

### SBWMV 19K protein is a conserved CRP

**Figure 1:**
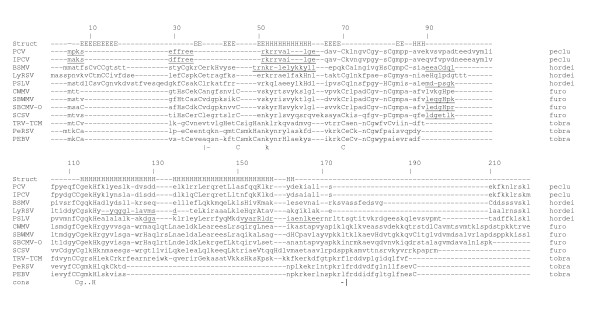
Amino acid sequence alignment of the CRPs encoded by furo-, peclu-, tobra- and hordeiviruses. The positions of amino acids are numbered above the alignment. The secondary structure prediction is shown directly above the alignment. Cys and His residues are bold uppercase letters. The leucines of leucine zippers are in bold face. The placement of residues that differ from Pfam are underlined. Vertical bars at the bottom represent where the Pfam family starts and stops. The genus for each virus is indicated on the right of the sequence. Abbreviations and accession numbers for the 33 aligned viruses are used (those displayed are underlined): LyRSV, *Lychnis ringspot virus *gi_1107721; CWMV-2, *Chinese wheat mosaic virus *gi_14270345; CWMV, gi_9635448; OGSV *Oat golden stripe virus*, gi_9635452; SBWMV-NE88 gi_9632360; SBWMV-NE gi_1449160; SBWMV OKl-1, gi_1085914; SBWMV-NY, gi_21630062; SBCMV-Ozz, *Soilborne cereal mosaic virus *gi_12053756; SBCMV-Fra, gi_9635249; SBCMV-O, gi_6580881; SBCMV-G, gi_6580877; SBCMV-C, gi_6580873; JSBWMV, *Japanese soilborne wheat mosaic virus *gi_7634693; SCSV, *Sorghum chlorotic spot virus *gi_21427644; PSLV, *Poa semilatent virus *gi_321642; BSMV-PV43, *Barley stripe mosaic virus *gi_19744921; BSMV-RUS, gi_94465; BSMV-JT, gi_808712; BSMV-ND18, gi_1589671; PCV, *Peanut clump virus *gi_20178597; IPCV, *Indian peanut clump virus *gi_30018260; TRV-PpK20, *Tobacco rattle virus*, gi_20522121; TRV-ORY gi_2852339; TRV-Pp085 gi_42733086; TRV-PSG, gi_112699; TRV-PLB, gi_465018; TRV-CAN, gi_1857116; TRV-FL, gi_3033549; TRV-RSTK, gi_6983830; TRV-TCM, gi_112701; PepRSV, *Pepper ringspot virus*, gi_20178602; PEBV, *Pea early browning virus*, gi_9632342.

The Pfam Protein Families Database reports a family of CRPs with similar sequences which includes proteins from BSMV, PSLV, PCV and SBWMV (Pfam 04521.5). Since there are viruses not included in the Pfam report that encode CRPs, this study was undertaken to determine if there is a larger CRP family containing related viral proteins. Further examination in this study reveals that the CRPs encoded by all known hordei-, peclu- and furoviruses share significant sequence similarity (Fig. 1). Efforts to find similarity between these proteins and CRPs encoded by pomo-, beny- and potyviruses were not successful. Whether these other plant viral CRPs are also suppressors of silencing can not be concluded at this point for two reasons: insufficient study and only weak sequence similarity relationships. Sequences of CRPs that affect virus replication and are encoded by members of other virus genera were also determined to be unrelated [25].

The SBWMV 19K protein is a CRP because it contains nine Cys residues [20]. Seven of these Cys residues are conserved in all furovirus proteins and are located in the N-terminal half of the protein. Five of these residues are within the block of sequences designated as protein family Pfam04521.5 and three of the conserved Cys residues are also conserved in the hordeiviral and pecluviral proteins. A selection from this alignment was corrected for several misplacements of short peptide sequences and is shown in Figure 1. The alignment represents the entire length of these proteins, although the termini are aligned with less confidence than the core. Examination of the tobraviral CRP sequences revealed sufficient similarity to justify their alignment with the Pfam04521.5 sequences. The alignment resulted in a significance score between 6 and 7, suggesting that the tobraviral proteins belong to this family.

The multiple sequence alignment of 33 CRPs from furo-, tobra-, peclu-, and hordeiviruses (Fig. 1) revealed three absolutely conserved residues: Cys70, Cys112, and His116 (numbering based on the aligned sequences). Gly113 was conserved in all viruses (except TRV-CAN) and is contained within a Cys-Gly-Xaa-Xaa-His motif in which one of the two Xaa residues is Lys or Arg. There is a Cys residue at position 7, 8 or 9 which is conserved in all except PCV and IPCV (pecluvirus) amino acid sequences. Alignment of the N-terminus is not exact since the PCV and IPCV proteins are N-terminally truncated. Within the N-terminal half, there are additional positions containing Cys residues that are conserved for some but not all viruses. For example, Cys9 is conserved among hordei-, tobra-, and some furoviruses; Cys at position 32 and 33 is conserved among all but pecluviruses; Cys36 is conserved among hordei- and furoviruses; Cys45 is conserved among furo and tobraviruses; Cys76 is conserved among furo and tobraviruses (except for SCSV; the pecluvirus PCV, but not IPCV, also has Cys76); Cys80 is conserved among all viruses except PeRSV and PEBV. Lys at position 52 and Arg at position 54 or 55 (Lys-Xaa-Arg or Lys-Xaa-Xaa-Arg) are conserved among all except PSLV. Gly at position 77 is conserved among all except tobraviruses.

The secondary structure prediction derived from the multiple sequence alignment is a long helical region extending from or slightly beyond the Cys-Gly-Xaa-Xaa-His motif to within 20 residues of the C-terminus. The furoviral proteins have spacings of conserved Leu residues from positions 89 to 106 consistent with a leucine zipper structure (which was not apparent in the original Pfam 04521.5). The N-terminal halves of the aligned amino acid sequences, containing most of the Cys residues, have a mixture of extended, helical and loop predicted structures.

The pecluviruses PCV and IPCV, and the hordeiviruses BSMV, LyRSV, and PSLV proteins contain a Ser-Lys-Leu sequence at the C-terminus. This tripeptide was shown for PCV to be a peroxisomal targeting signal [16]. This signal is not present in CRPs of furo- or tobraviruses.

### SBWMV 19K CRP aggravates PVX-induced symptoms

**Figure 2:**
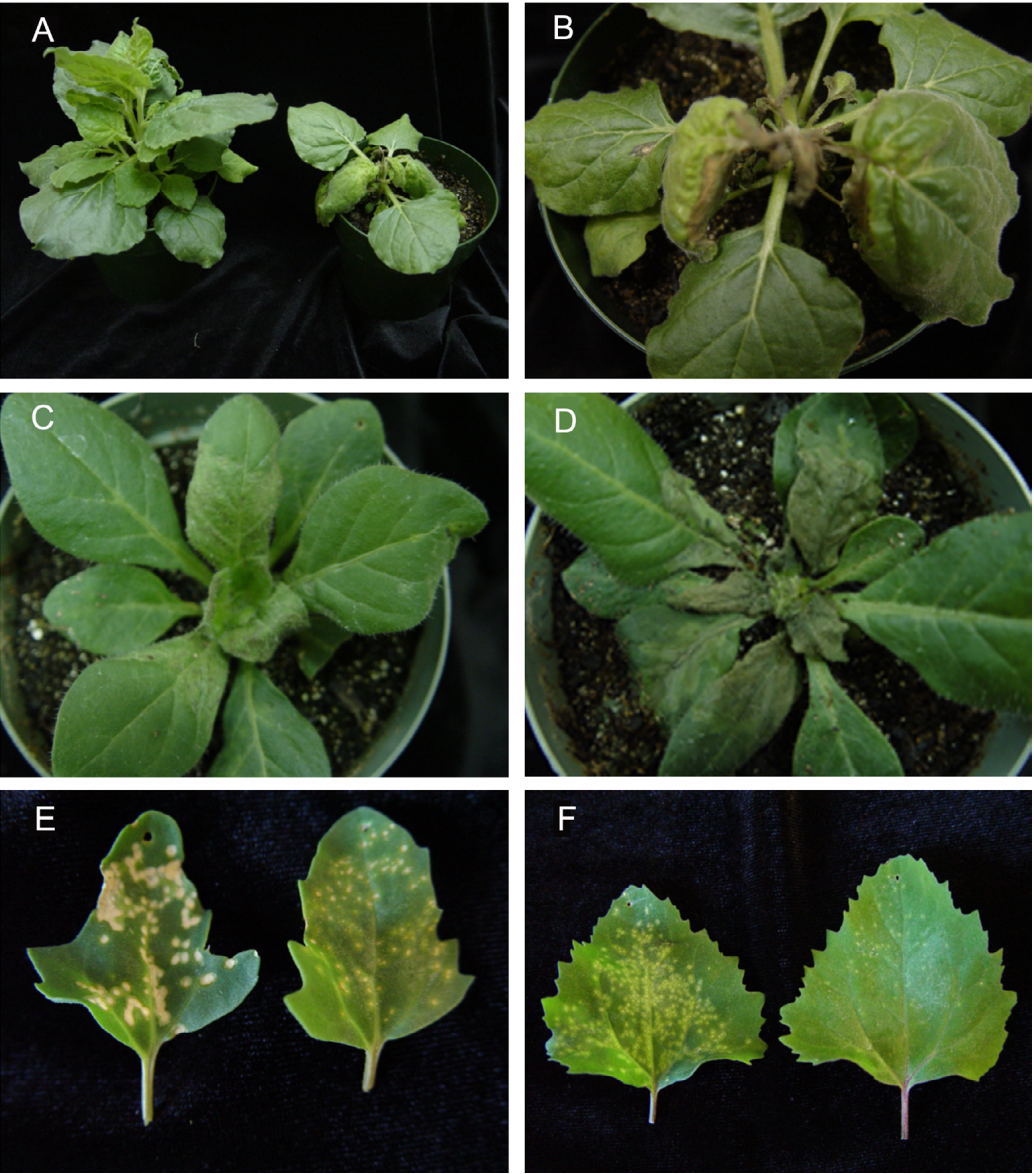
Plants infected with PVX.GFP or PVX.19K at 21 dpi. **(A) ***N. benthamiana *plants infected with PVX.GFP (left) and PVX.19K (right). **(B, D) **PVX.19K-infected *N. benthamiana *and *N. clevelandii *plants, respectively, at 21 dpi show systemic necrosis. **(C) **PVX.GFP-infected *N. clevelandii *plants. **(E, F) ***C. quinoa *and *C. amaranticolor *leaves infected with PVX.19K (left both panels) and PVX.GFP (right in both panels).

The tobravirus and hordeivirus CRPs have been described as pathogenicity determinants that regulate symptom severity in infected plants [15]. Since the SBWMV 19K protein is a similar CRP, experiments were conducted to determine if it also has an effect on symptom expression. The SBWMV 19K ORF was inserted into the PVX genome and PVX.19K infectious transcripts were used to inoculate *N. benthamiana, N. clevelandii, C. quinoa*, and *C. amaranticolor *leaves (Fig. 2). As a control, plants were also inoculated with PVX.GFP, which has the green fluorescent protein (GFP) gene inserted into the viral genome. The spread of PVX.GFP expression was monitored using a handheld UV lamp to monitor GFP expression and verify systemic virus accumulation (data not shown).

Symptoms were first observed in plants inoculated with PVX.GFP and PVX.19K between 10 and 14 dpi. By 21 dpi, systemic necrosis was evident in *N. benthamiana *and *N. clevelandii *plants inoculated with PVX.19K (Fig. 2A, B and 2D) while PVX.GFP infected plants showed systemic mosaic symptoms (Fig. 2A and 2C). *N. benthamiana *plants infected with PVX.19K were clearly stunted in comparison to plants infected with PVX.GFP (Fig. 2A). The PVX.19K infected *N. clevelandii *leaves collapsed by 21 dpi (Fig. 2D).

**Figure 3:**
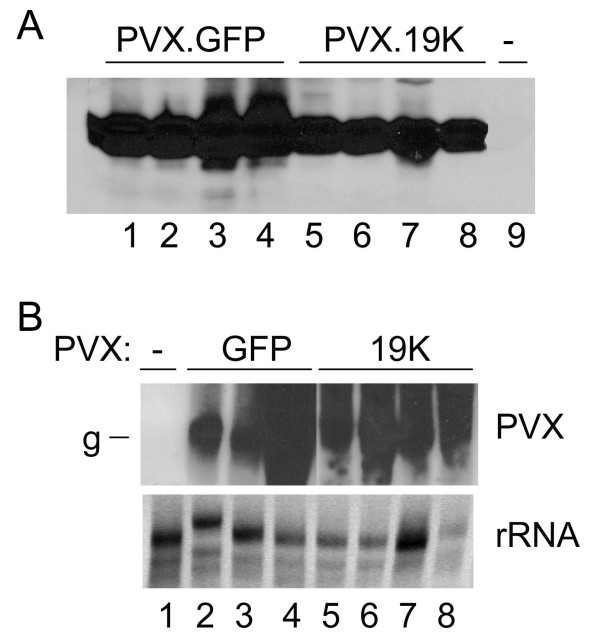
Immunoblot and northern analyses of the PVX infected *N. benthamiana *plants. **(A) **Immunoblot analysis conducted using PVX CP antiserum show similar levels of PVX.GFP virus (lanes 1–4) and PVX.19K virus (lanes 5–8). Lane 9 contains extract of non inoculated plants. **(B) **Northern analysis of RNA isolated from a healthy plant (lane 1), upper noninoculated leaves of PVX.GFP infected plants (lanes 2 – 4) and upper noninoculated leaves of PVX.19K infected plants (lanes 5 – 8). Blots were probed with a GFP sequence probe. The bottom image is the ethidium bromide stained gel showing ribosomal RNAs. Abbrev.: g, genomic RNA.

Immunoblot and northern analyses were conducted to verify PVX accumulation in the upper leaves of *N. benthamiana *plants. Immunoblot analysis was conducted using anti-PVX CP serum. High levels of PVX CP was detected in plants that were systemically infected with PVX.GFP (Fig. 3A lanes 1–4) and PVX.19K (Fig. 3A lanes 5–8). The SBWMV 19K CRP had no obvious effect on PVX accumulation in upper noninoculated leaves. Viral RNA accumulation was analyzed by northern blot and high levels of genomic RNA was detected in the upper leaves of PVX.GFP (Figure 3B lanes 2–4) and PVX.19K (Fig. 3B lanes 5–8) inoculated plants. Thus, the SBWMV 19K CRP did not seem to have a deleterious effect on PVX accumulation. RT-PCR was used to verify that the SBWMV 19K ORF was maintained in the PVX genome in systemically infected plants. RNA samples taken from the upper leaves of *N. benthamiana *plants which were used for northern analysis, were also used in RT-PCR reactions to verify the presence of the SBWMV 19K ORF in the PVX genome. In all samples it appeared that the SBWMV 19K CRP was stably maintained in the PVX genome (data not shown).

PVX.19K produced large necrotic lesions in the *C. quinoa *and *C. amaranticolor *leaves. Local lesions were detected in plants inoculated with PVX.GFP or PVX.19K between 5 and 7 dpi. PVX.19K-inoculated *C. quinoa *plants showed severe necrotic local lesions (Fig. 2E). The necrotic lesions gradually merged and the infected tissue eventually collapsed. PVX.19K-inoculated *C. amaranticolor *leaves showed enlarged chlorotic lesions advancing to necrotic lesions over time (Fig. 2F). PVX.GFP-inoculated *C. quinoa *leaves showed small chlorotic and necrotic local lesions while PVX.GFP-inoculated *C. amaranticolor *leaves showed mild flecks (Fig. 2F). Association of PVX.GFP with the local lesions was verified using a hand held UV lamp (data not shown).

### SBWMV 19K CRP is a suppressor of RNA silencing

**Figure 4:**
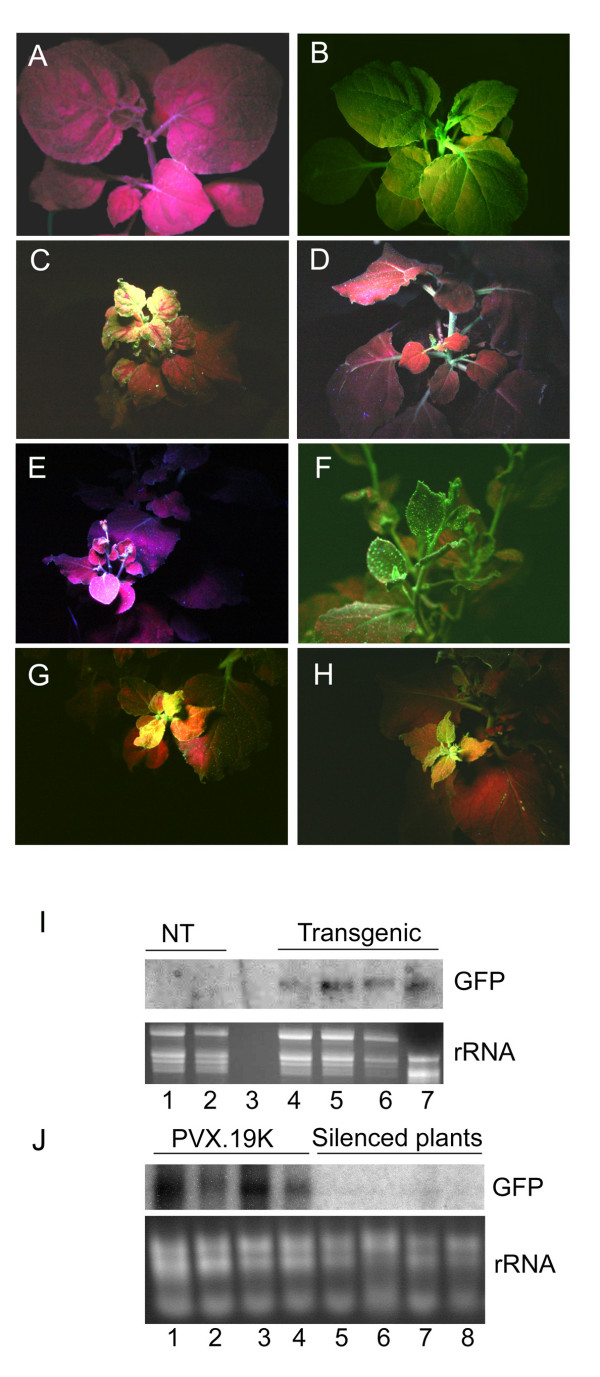
Evidence for RNA silencing suppression by the SBWMV 19K CRP. **(A) **nontransgenic *N. benthamiana *under a UV lamp exhibits red fluorescence due to chlorophyll. **(B) **GFP-transgenic *N. benthamiana *(line 16C) exhibits green fluorescence under a UV lamp. **(C) **GFP was systemically silenced in the 16C transgenic *N. benthamiana *following infiltration with *Agrobacterium*. Here in the upper most leaves GFP silencing is vein centric. Systemic GFP silencing is detected initially within 2 weeks. **(D) **Within 3 weeks, GFP expression is completely silenced in the upper leaves. **(E) **GFP silenced plant inoculated with PVX.GUS. Emerging tissues of the infected plant remain silenced. **(F, G, and H) **GFP expression was observed in the emerging tissues of plants that were inoculated with PVX.19K. **(I) **Northern analyses of total RNAs from nontransgenic tissues (lanes 1, 2) and GFP transgenic tissues (lanes 4 – 7) probed with a labeled GFP sequence probe. Lane 3 is blank. Lanes under the northern blot show ribosomal RNAs on an ethidium bromide stained gel. **(J) **Northern analysis of total RNAs from 16C plants infiltrated with Agrobacterium containing GFP constructs and probed with a labeled GFP sequence probe. Lanes 1–4 are RNA samples taken from plants that were also inoculated with PVX.19K. Lanes 5–8 are RNA samples taken from plants inoculated with PVX.GUS. Lanes under the northern blot show ribosomal RNAs.

In this study we employed a widely used "reversal of silencing assay" to determine if the SBWMV 19K CRP is a suppressor of RNA silencing in plants [28]. In this assay, GFP-expression in the 16C transgenic *N. benthamiana *plants (Fig. 4B) was silenced by infiltrating young leaves with a suspension of *Agrobacterium *expressing GFP. The progression of GFP silencing was viewed first locally and then systemically using a hand held UV lamp. Within two weeks, the spread of GFP silencing was viewed systemically (Fig. 4C) and by three weeks, the only visible fluorescence is red fluorescence due to chlorophyll (Fig. 4D). At this time, the silenced plants were inoculated with PVX.19K. As PVX.19K viruses spread locally and then systemically, there was no change in GFP expression in the inoculated leaves or in the upper leaves (Fig. 4E). However, GFP expression was observed in the emerging leaves (Fig. 4F – H). The SBWMV 19K CRP prevented RNA silencing only in emerging leaves where RNA silencing had not developed prior to virus infection. As a control, plants were also inoculated with PVX.GUS following infiltration with *Agrobacterium*. There was no evidence of GFP expression in the inoculated, mature, or new emerging leaves. The silencing phenotype was unaffected by PVX.GUS.

Northern analyses was conducted to confirm RNA silencing in the upper leaves of *Agrobacterium*-infiltrated leaves and in the plants inoculated with PVX.GUS (Fig. 4I and 4J). GFP specific RNAs were detected in transgenic plants (Figure 4I lanes 4–7) and emerging leaves of plants injected with *Agrobacterium *and inoculated with PVX.19K (Figure 4J lanes 1–4). GFP specific RNAs were not detected in untreated nontransgenic plants (Figure 4I lanes 1–3) or in plants that were injected with *Agrobacterium *and inoculated with PVX.GUS (lanes 4–8). RNA samples collected from non silenced and silenced plants were also tested by Northern analysis to confirm the systemic accumulation of PVX.GUS or PVX.19K (data not shown). Since, GFP expression was restored in plants systemically infected with PVX.19K but remained silenced in plants inoculated with PVX.GUS, it is likely that the SBWMV 19K ORF is a suppressor of RNA silencing.

## Discussion

Many viruses encode proteins that suppress RNA silencing but the phylogenetic relatedness of these proteins is poorly understood. In this study, one class of viral CRPs, which were described as suppressors of RNA silencing and/or viral pathogenicity determinants, were shown to be phylogenetically related. These CRPs have a conserved Cys-Gly-Xaa-Xaa-His motif in which one of the two Xaa residues is Lys or Arg. The N-terminus has several conserved Cys residues that likely comprise a zinc finger motif. In fact, the ability of the gamma b protein of BSMV to bind Zn(II) was recently demonstrated [25].

Prior to 1999, SBWMV, BNYVV, PCV, and PMTV belonged to the genus *Furovirus*. As sequence data from different furoviruses have become available, it became clear that there are significant differences in the genome organization of these viruses, and therefore furovirus classification was revised in 1999 [19]. The genus *Furovirus *now consists of viruses similar in genome organization to SBWMV [29]. These viruses are bipartite and have a single MP that is phylogenetically related to the tobamovirus and dianthovirus MPs [20,22]. BNYVV, PCV, and PMTV were reclassified into the genera *Benyvirus, Pecluvirus*, and *Pomovirus*, respectively, for two reasons [19,29]. First, the MPs of these viruses are phylogenetically distinct from SBWMV. BNYVV, PCV, and PMTV each possess a cluster of three slightly overlapping ORFs known as the "triple gene block", which has been shown for BNYVV [30] to mediate viral cell-to-cell movement. Second, benyviruses and pomoviruses differ from furoviruses in the number of genome segments. BNYVV has four or five genome segments while PMTV has three genome segments [31]. Pecluviruses like furoviruses have two genome segments, thus the primary difference between these virus genera is the MP ORFs [32]. This is significant because the initial amino acid sequence comparisons of CRPs from furo-, hordei-, tobra-, and carlaviruses included BNYVV as the type-member of the genus *Furovirus *and concluded that these small CRPs were unrelated [33]. Reclassification of the BNYVV as a member of the genus *Benyvirus *and inclusion of new members into the genus *Furovirus *led us to reexamine the relatedness of the viral CRPs. Based on the most recently defined taxonomic structure, the current amino acid sequence comparison presented in Figure 1 indicates that the CRPs derived from viruses of the genera *Furo-, Hordei-, Peclu*-, and *Tobravirus *are phylogenetically related. On the other hand, these proteins are so different from CRPs encoded by *Pomo*-, *Beny- *and *Carlaviruses *that the latter ones could not be included in the alignment (Fig 1).

The present study shows that the SBWMV 19K CRP, when expressed from the PVX genome, functions as a pathogenesis factor and a suppressor of RNA silencing. The SBWMV 19K CRP, when it was expressed from the PVX genome, induced systemic necrosis on *Nicotiana benthamiana, N. clevelandii, C. quinoa*, and *C. amaranticolor*. These symptoms are distinct from the symptoms associated with PVX infection in these hosts, and from symptoms induced by SBWMV in its natural hosts. In systemic hosts, both PVX and SBWMV typically cause mosaic symptoms that range from mild to severe. In *C. quinoa *and *C. amaranticolor *both PVX and SBWMV cause mild chlorosis. Severe necrosis and ultimate collapse of the tissue has been reported for other unrelated viral proteins that are pathogenicity factors and suppressors of RNA silencing. This include the *Poa semilatent virus *(PSLV) gamma b, TBSV P19, *Tobacco etch virus *HC-Pro, and the *Rice yellow mottle virus *P1 proteins[7,11,14,34].

When we introduced the SBWMV 19K ORF into the TBSV vector and inoculated it to *N. benthamiama, N. tabacum, C. quinoa*, and *C. amaranticolor *(data not shown) plants, the SBWMV 19K CRP did not have any effect on symptomology (data not shown). However, it was reported previously that protein expression levels from the TBSV vector might be too low to test the effects of heterologous proteins on symptom severity [35]. Since an antibody to the SBWMV 19K CRP is unavailable, the levels of protein expression from PVX or TBSV vectors could not be analyzed to determine if gene dosage or protein expression levels contribute to symptom severity.

In a related study, the SBWMV 19K and the BSMV gamma b CRPs could substitute for the TRV 16K CRP within the TRV genome, promoting virus replication and systemic accumulation [15]. The ability of the SBWMV 19K and the BSMV gamma b CRPs to induce severe symptoms when expressed from the PVX genome is reminiscent of phenomena described in relation to viral synergisms. The best studied viral synergism is between *Tobacco etch virus *(TEV) and PVX in which the TEV HC-Pro protein enhances accumulation and disease severity of PVX [34]. HC-Pro promotes infection of PVX by suppressing the anti-viral RNA silencing defense mechanism that would normally act on PVX to reduce virus infection. HC-Pro has the ability to increase PVX accumulation in the same way the SBWMV 19K CRP and the BSMV gamma b proteins were shown previously to enhance accumulation of TRV in infected plants [15].

## Conclusion

The phylogenetic relatedness of the hordei-, peclu-, and furovirus CRPs is further substantiated by evidence that these proteins are all capable of suppressing RNA silencing in emerging leaves. This was demonstrated in the present and related studies using the same reversal of silencing assay used in this study. The SBWMV 19K CRP, the BSMV and PSLV gamma b CRPs, and the PCV 15K CRPs were each unable to change GFP expression in leaves where GFP was silenced prior to virus infection. However in each case, GFP expression occurred in newly emerging leaves [14,16]. Thus, members of this family of CRPs similarly act on the RNA silencing machinery to block spread of the silencing signal into newly emerging leaves. In each case, the silencing suppressor activities of these CRPs have been compared to CMV and potyviruses in preventing onset of RNA silencing in new growth [14,16]. While there is no evidence that the hordei-, peclu- and furovirus CRPs are related to the CMV or potyvirus silencing suppressors, it seems that the mode of action might be conserved among diverse viruses.

## Methods

### Amino acid sequence comparisons

Related protein sequences were identified and retrieved from the NCBI data bank using PSI-BLAST. A PSI-BLAST search was launched with the amino acid sequence of the 19K CRP of *Chinese wheat mosaic virus *(CWMV, a furovirus). A similar search began with the amino acid sequence of BSMV gamma b, a sequence recovered in the CWMV search. Both searches converged at the second iteration and retrieved the same set of 22 sequences. This set contained CRPs derived from furo-, peclu- and hordeiviruses and contained the conserved P18 PFAM domain ("protein family"URL reference [36].

A preliminary alignment of the retrieved proteins sequences was performed using the multiple sequence alignment mode of ClustalX. These twenty two furovirus and hordeivirus sequences were aligned using ClustalX alignments suggested in the BLAST outputs and PFAM. The tobraviral CRPs were not recovered by the above procedure, but upon manual inspection, appeared to have Cys residues in a linear arrangement that was similar to the set of 22 proteins. Eleven tobraviral protein sequences, exclusive members of a conserved domain in the Conserved Domain database  were aligned using ClustalX [37]. This tobraviral amino acid sequence alignment and the alignment of the 22 amino acid sequences sequences were assembled by ClustalX in profile mode, followed by manual adjustment. Amino acid sequences of aligned furo- and hordeiviral proteins were aligned with tobraviral amino acid sequences in profile mode of ClustalX (a total of thirty three sequences were aligned). A total of 33 amino acid sequences were aligned. In all cases, adjustments to the alignments were made using Se-Al [38]. Significance scores for the alignment of the two groups of protein sequences were calculated as previously described, using a structural conservation matrix, SCM2, for scoring [39].

### Plasmids and bacterial strain

All plasmids were used to transform *Escherichia coli *strain JM109 [40]. The plasmids pPVX.GFP is an infectious viral clone and contains a bacteriophage T7 promoter [39]. The pPVX.GFP plasmid contains the PVX genome and the GFP adjacent to a duplicated CP subgenomic promoter. The plasmid pHST2-14 contains the TBSV genome and a mutation in the TBSV P19 ORF eliminating expression of a protein that suppresses RNA silencing [10,42]. The plasmid pTBSV.GFP contains GFP inserted into the TBSV genome replacing the viral CP ORF [10].

The SBWMV 19K CRP ORF was inserted into the PVX.GFP genome, replacing the GFP ORF. The 19K CRP ORF was reverse transcribed and PCR amplified from purified SBWMV RNA using a forward primer (GCG GGG ATC GAT ATG TCT ACT GTT GGT TTC CAC) containing added sequences encoding a *Cla*I restriction site (underlined) and a reverse primer (CGC GTC GAC TCA CAA AGA GGA TAT CTT CTT TGG C) containing sequences encoding a *Sal*I restriction site (underlined). PCR products and pPVX.GFP plasmids were digested with *Cla*I and *Sal*I and then were ligated to prepare pPVX.19K.

### In vitro transcription and plant inoculations

*In vitro *transcription reactions contained: 0.25 μg of linearized DNA, 5 μl of 5X T7 transcription buffer, 1.0 μl of 0.1 M DTT, 0.5 μl of SUPERase·In™ ribonuclease inhibitor (20 U/ μl) (Ambion, Austin, TX), 2.5 μl of an NTP mixture containing 5 mM ATP, CTP, UTP, and GTP (Pharmacia-Pfizer, Mississauga, Ontario, Canada), 0.7 μl of T7 polymerase (Ambion), and nuclease-free water to a final volume of 25 μl. The reactions were incubated for one and a half hour at 37°C [10].

*Nicotiana benthamiana, N. clevelandii, Chenopodium quinoa*, and *C. amaranticolor *plants were inoculated with infectious transcripts to study disease severity. Four plants, two leaves per plant, were inoculated in each experiment. Experiments were repeated at least three times. Ten μl of undiluted PVX.GFP or PVX.19K transcripts were rub-inoculated to each plant.

The transgenic *N. benthamiana *line 16C was used to study RNA silencing. This line is homozygous for the GFP transgene at a single locus [44]. Plants were inoculated with transcripts following infiltration with *Agrobacterium *(see below).

### Agrobacterium infiltration of leaves

*Agrobacterium tumefaciens *strain C58C1 (pCH32) carrying a binary plasmid expressing GFP from a *Cauliflower mosaic virus *(CaMV) 35S promoter was used to silence GFP expression in *N. benthamiana *line 16C. *Agrobacterium *cultures were grown overnight at 28°C in 5 ml of L-broth medium containing 5 μg/ml of tetracycline and 50 μg/ml of kanamycin. This 5 ml culture was used to inoculate 50 ml L-broth and grown overnight in medium containing 5 μg/ml tetracycline, 50 μg/ml kanamycin, 10 mM MES, and 20 μM acetosyringone. Cultures of *Agrobacterium *containing GFP were pelleted by centrifugation and resuspended in a solution containing 10 mM MgCl_2_, 10 mM MES, and 150 μM acetosyringone. The final concentration of *Agrobacterium *was 0.5 OD_600_. The suspension was left at room temperature for 2–3 hours and then loaded into a 2 ml syringe. The syringe was used to infiltrate the suspension into the underside of the leaf.

### Visualization of GFP

A hand-held model B-100 BLAK-RAY long wave ultraviolet lamp (Ultraviolet Products, Upland, CA) was used to monitor GFP expression in 16C plants infiltrated with *Agrobacterium *and in PVX.GFP inoculated plants. GFP fluorescence was recorded with a Sony Digital Still Camera model DSC-F717 (Sony Corporation of America, New York City, New York). In all plants analyzed, GFP expression was monitored every 3 days for up to 21 days post inoculation (dpi) or post infiltration with *Agrobacterium*.

### Immunoblot analysis

Immunoblot analyses were conducted according to [40]. Total protein from uninfected and infected *N. benthamiana *leaves was extracted in 1:10 (w/v) grinding buffer (100 mM Tris-HCl pH 7.50, 10 mM KCl, 5 mM MgCl_2_, 400 mM sucrose, 10% glycerol, and 10 mM β-mercaptoethanol). Extracts were centrifuged at 10,000 g for 10 min. Equal volumes of supernatants and protein loading buffer (2 % SDS, 0.1 M dithiothreitol, 50 mM Tris-HCl pH 6.8, 0.1% bromophenol blue, and 10 % glycerol) were mixed and boiled for 5 min. SDS-PAGE was carried out for 1 h at 200 V using 30 μl of each sample and 12.5% SDS -PAGE and the Biorad Mini-Protean 3 system (Biorad Laboratories, Hercules, CA). Proteins were transferred to PVDF membranes (Amersham Biosciences Corp., Piscataway, NJ) at 4°C overnight using protein transfer buffer (39 mM glycine, 48 mM Tris base, 0.037% SDS, and 20% methanol, pH 8.3) and a BioRad Trans-Blot system (BioRad Laboratories). Immunoblot analyses were conducted using the ECL-Plus Western Blotting Detection Kit (Amersham Biosciences Corp.). PVX CP antiserum (1:200) (Agdia, Elkhart, IN) was used.

### Northern analysis

Northern analyses were conducted according to [40]. For analyses of PVX infected plants and GFP expressing transgenic plants, a radiolabeled DNA probe was prepared using Rediprime II Random Prime Labeling System (Amersham Biosciences Corp.). Labeling was conducted using PCR products corresponding to either the GFP or PVX CP ORFs.

For detection of TBSV.GFP and TBSV.19K in infected plant extracts, a DNA probe was labeled with digoxigenin (DIG). TBSV.GFP plasmids were digested with *Nco*I and *Sal*I and a 614 nt fragment was gel eluted and labeled using Dig High Prime kit (Roche Applied Science Inc. Indianapolis, IN). The CSPD DIG Luminescence Detection Kit (Roche Applied Science Inc.) was used for chemiluminescence detection of DIG-labeled probes. Special thanks to Wenping Qui at Southwest Missouri State University for assistance with studies using TBSV to express the SBWMV 19k.

The p26SBE-2 plasmid was obtained from Kay Scheets at Oklahoma State University and contains the 26S ribosomal RNA gene in pBluescript. This plasmid was used to prepare a DNA probe for membrane detection of rRNA [45]. The p26SBE-2 plasmid was digested with *Bam*HI and *Eco*RI and a 1 kb fragment corresponding to the 26S rRNA was recovered and labeled using the Dig High Prime DNA labeling system (Roche Applied Science Inc.).

## Competing interests

The author(s) declare that they have no competing interests.

## Authors' contributions

Jeannie Te did all cloning, plant inoculation experiments, gene silencing experiments. Ulrich Melcher did the amino acid sequence alignments and phylogenetic comparisons. Amanda Howard did some gene silencing experiments, photography. Jeanmarie Verchot-Lubicz conceived the study, did some molecular analysis, and wrote the paper. Special thanks to Wenpiny Qiu at Southwest Missouri State University for assistance with studies using TBSV to express the SBWMV 19k.
